# Conservative approach to a rare case of persistent sciatic artery with iatrogenic femoral arteriovenous fistula: a case report

**DOI:** 10.3389/fsurg.2025.1498368

**Published:** 2025-02-26

**Authors:** Lizhao Wang, Yan Gu, Sen Yang

**Affiliations:** Department of Vascular Surgery, Tianjin First Central Hospital, Tianjin, China

**Keywords:** persistent sciatic artery, dysplasia, iatrogenic arteriovenous fistula, common femoral vein, superficial femoral artery

## Abstract

Persistent sciatic artery (PSA) is a rare anatomic variant disease with an incidence of approximately 0.025%–0.05% it is considered to be an axial congenital vascular malformation, which may be related to the failure of sciatic artery degeneration and iliofemoral artery dysplasia. Some patients may be asymptomatic, while others experience chronic pain, thrombosis, and aneurysm formation. We report the case of a 63-year-old female patient with a superficial femoralartery (SFA)-common femoral vein (CFV) arteriovenous fistula found on ultrasound of the lower extremities due to soreness and numbness of the lower limbs. Interventional surgery and computed tomography were performed to close the internal fistulas and detect the PSA. A coated stent graft was inserted to close the arteriovenous fistula. After 1-mongth follow-up, lower limb discomfort disappeared, and she was administered symptomatic treatment with anticoagulation and swelling reduction medication. Six months after surgery, computed tomography indicated the disappearance of internal fistula and good PSA progression. Lower extremity symptoms were considered to be related to the femoral arteriovenous fistula; therefore, no intervention was performed for the PSA, we only provided health education, such as reducing sedentary and right lying to prevent vascular lesions.

## Introduction

The sciatic artery originates from the umbilical artery and is the main source of blood supply to the lower extremities during embryonic development. The sciatic arteries eventually recede, leaving behind remnants that persist as the popliteal and peroneal arteries. Prior to sciatic artery degeneration, the popliteal and peroneal arteries establish continuity with the superficial femoral artery (1, 2). If degeneration fails, it formed a persistent sciatic artery (PSA), which is a direct continuation of the internal iliac artery and may cause serious complications, such as chronic pain, thrombosis, and aneurysm formation. It is a rare anatomical variant with an incidence of approximately 0.025%–0.05% (3). Cases of PSA combined with arteriovenous fistula are very rare in the literature retrieved so far.

## Case report

The patient was a 63-year-old woman with a history of paroxysmal atrial fibrillation for 20 years, who had undergone radiofrequency ablation surgery via femoral artery puncture twice in the Department of Cardiology of our hospital, and had been diagnosed with hypertension 1 month prior. Clopidogrel was administered for antiplatelet treatment. Fifteen days prior, she was re-examined in the cardiology department after complaining of a feeling of heaviness and soreness in the lower extremities with mild swelling. Ultrasound of the lower limb blood vessels revealed a femoral arteriovenous fistula, due to which she was admitted to our department (Table 1).

**Table 1:** Care-related timeline.

Timeframe	Events & findings
Last 20 years	A history of paroxysmal atrial fibrillation.
Last month	Underwent femoral-artery puncture radiofrequency ablation twice in our hospital's Cardiology dept. Received clopidogrel for anti-platelet therapy.
15 days ago	Re-examined in the cardiology department after complaining of lower limb soreness.
15 days ago	Ultrasound of the lower extremity vessels showed the formation of a fistula in the right superficial femoral artery-right common femoral vein.
Post-hospital admission	Computed tomography indicated the formation of the fistula, then we find a special artery originated from the right internal iliac artery, continued along the popliteal artery and the inferior knee artery downward, while the right SFA originated from the CFA and the right external iliac artery, then suddenly narrowed and disappeared in the middle of the thigh. Hypoplasia of the SFA was detected, along with the right PSA.
Post-admission to our department	Angiography was performed to confirm the presence of the PSA and the fistula. A covered stent-graft (8 mm*2.5 cm) was implanted in the SFA. Anticoagulation and swelling reduction medication were given.
Post-operation	Angiography indicated the disappearance of the fistula. Lower limb discomfort symptoms were reduced
1 Month post-operation	The lower limb-related symptoms disappeared.
6 Month post-operation	No symptoms of any lower limb discomfort. computed tomography indicated the disappearance of internal fistula and anatomically well-run PSA.

Physical examination revealed that the patient had a well-developed, symmetrical, and normal gait. The bilateral femoral, popliteal, dorsal, and posterior tibial artery pulses were palpable, and the right femoral artery showed a palpable bruit. The skin of both lower limbs was warm; the colour, muscle strength, sensation, and movement of both lower limbs were normal; there was no oedema; and no abnormalities were observed on neurological examination.

Laboratory tests after admission (routine blood tests, biochemistry, blood coagulation function) did not reveal any obvious abnormalities. Ultrasound of the lower extremity vessels showed the formation of a fistula in the right superficial femoral artery-right common femoral vein.

**Figure 1:**
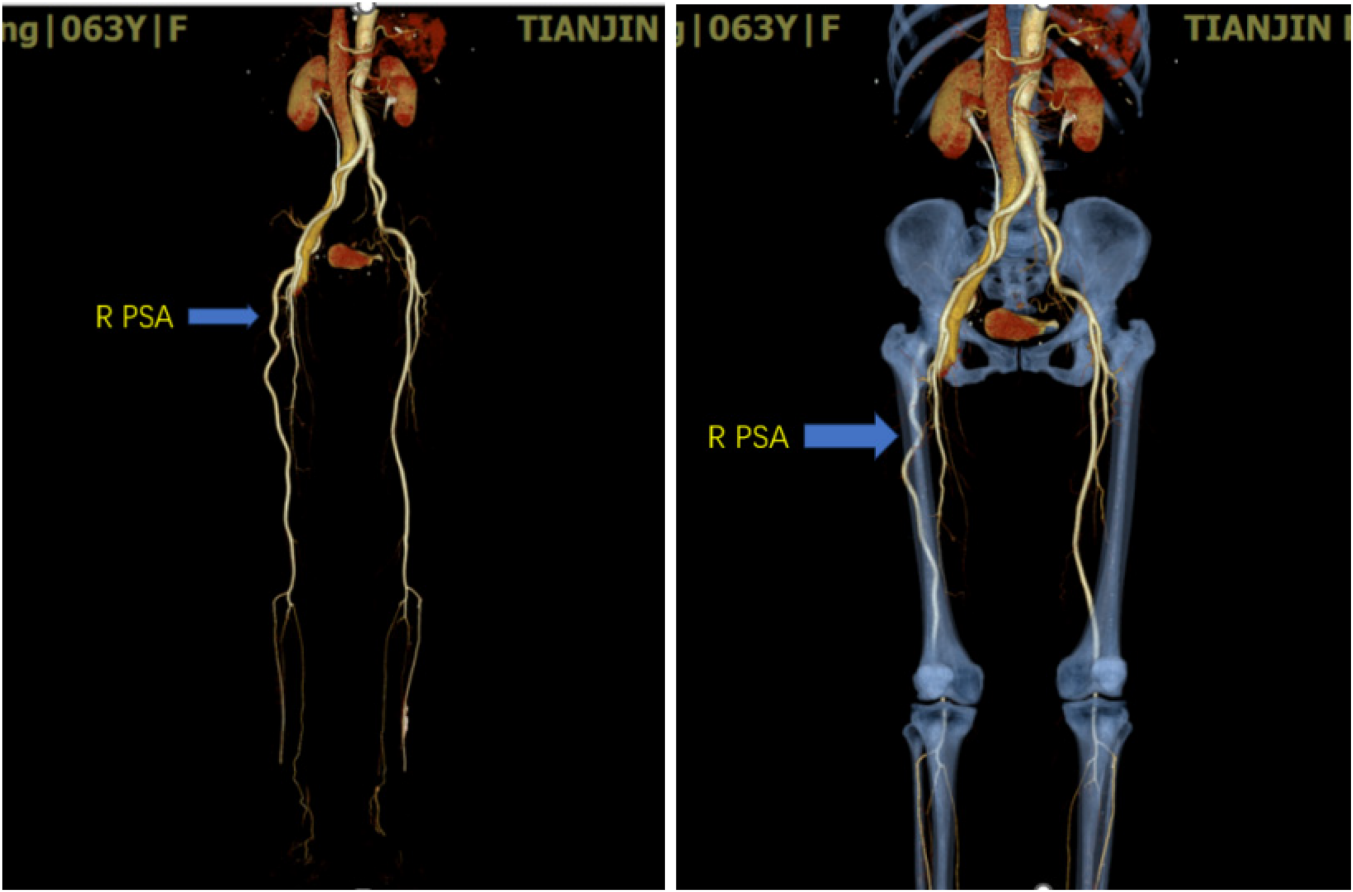
Three-dimensional reconstruction of the lower extremity arteries. The arrow in the diagram indicates the persistent sciatic artery.

**Figure 2:**
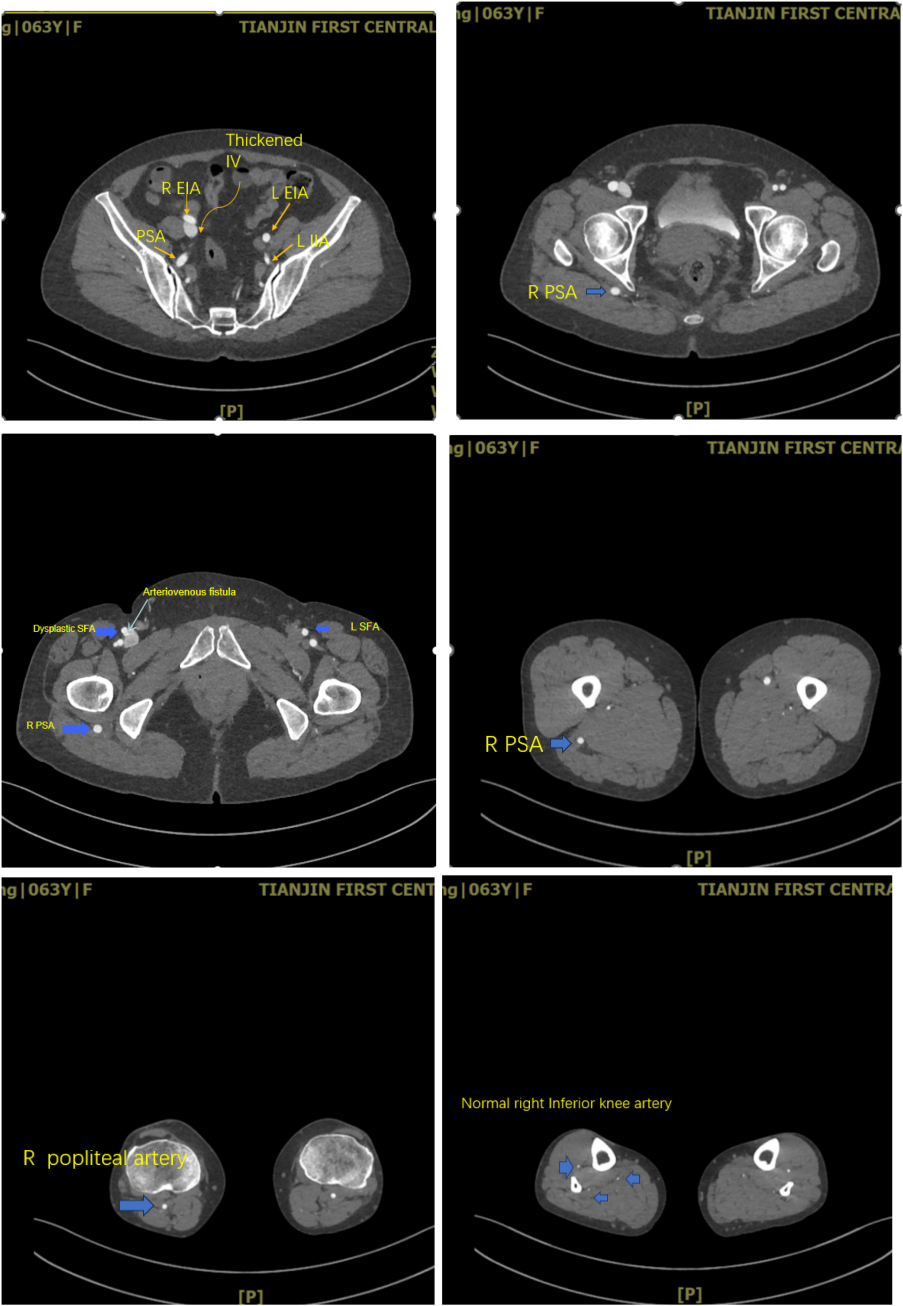
This series of pictures shows the running of the right persistent sciatic artery.

After being admitted to the hospital, Computed tomography angiography of the lower limbs confirmed the formation of the right superficial femoral artery-right common femoral venous fistula. Hypoplasia of the right SFA was detected, along with the right PSA (Figures 1, 2). The PSA originated from the right internal iliac artery, continued along the popliteal artery and the inferior knee artery downward, while the right SFA originated from the right CFA and external iliac artery, and suddenly narrowed and disappeared in the middle of the thigh.

**Figure 3:**
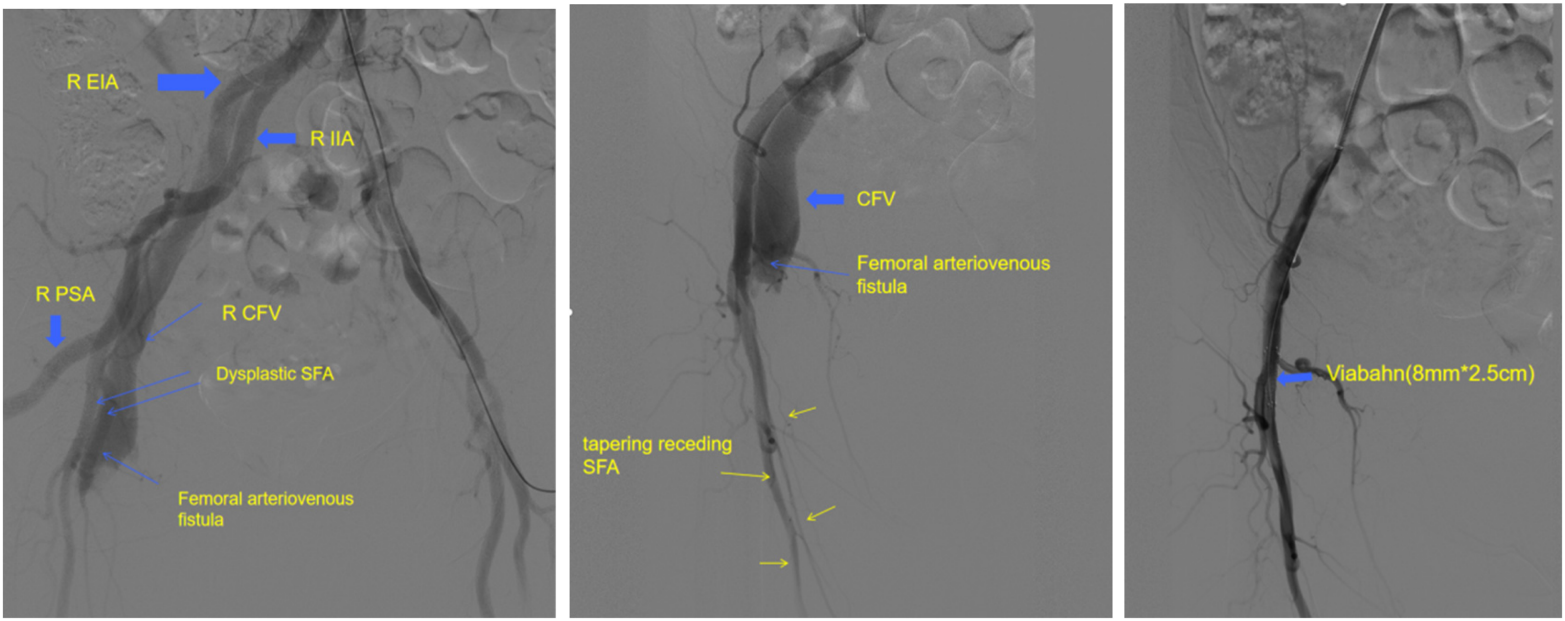
Arteriovenous fistula was seen on angiographic surgery. The PSA was confirmed intraoperatively. We placed a covered stent-graft to seal the internal fistula.

**Figure 4:**
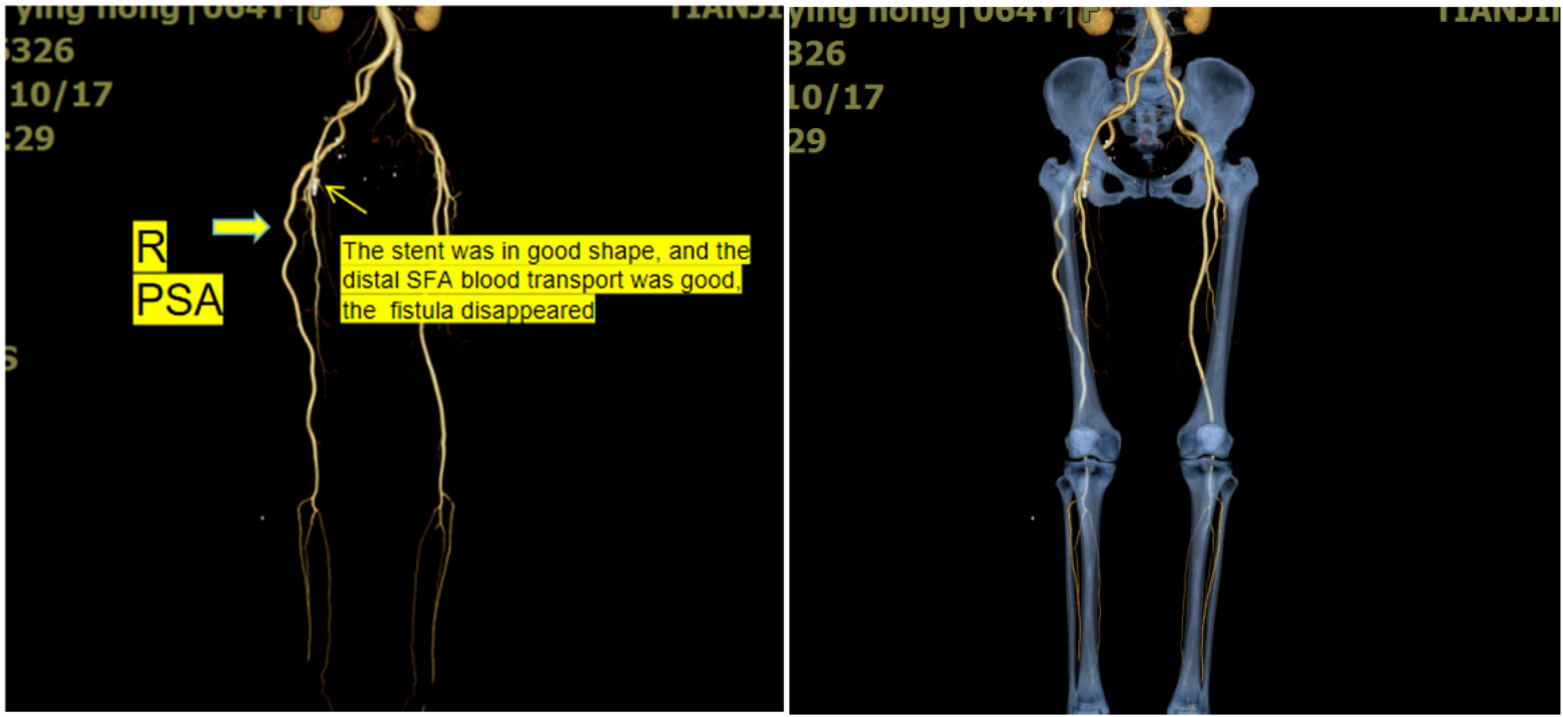
This series of pictures shows the CT imaging findings at 6-month follow-up.

Angiography was performed to confirm the presence of PSA (Figure 3). Due to the history of recent interventional surgery, the internal fistulae was considered iatrogenic. Considering that the patient's lower limb symptoms were related to arteriovenous fistula while not related to PSA, we closed the right arteriovenous fistula by implanting a covered stent-graft (8 mm*2.5 cm) in the superficial femora. Postoperative angiography revealed the disappearance of the arteriovenous fistula. The patient was advised to avoid sitting for a long time, and was administered symptomatic treatment with anticoagulation and swelling reduction medication. She was advised to follow up regularly in the vascular surgery clinic. The lower limb-related symptoms disappeared after 1 month. Six months after surgery, computed tomography indicated the disappearance of internal fistula and anatomically well-run PSA. The patient had normal cardiac function, normal dorsal foot/posterior tibial artery beats, warm skin, and no significant sensory and motor abnormalities (Figure 4). Quality of life was significantly improved compared to preoperative period.

## Discussion

In 1832, Green first reported the presence of a persistent sciatic artery in a postmortem case (4). PSA is a rare anatomical variant that persists in adulthood and predisposes patients to complications such as aneurysms and distal limb-threatening thromboembolism. PSA occurs bilaterally, with no significant differences according to sex or side of the body (5, 6). The incidence is approximately 0.025%–0.05 percent. Van Hooft et al. reported that the mean age at diagnosis of PSA was 57 years, with an almost equal sex distribution; according to a review of the literature, 56% of patients were female and 44% were male. Unilateral PSA accounts for 70% PSA cases (7). The rate of aneurysm formation in patients with PSA is approximately 44%.

Anatomically, the PSA is the continuation of the internal iliac artery. It accompanies the sciatic nerve, passes through the ischial foramen, and follows the back of the leg all the way to the ankle. Gauffre classifies the PSA into 5 categories. In types 1 and 2, PSA is fully present (8). The difference between types 1 and 2 is that in type 1, there is a fully developed SFA, whereas in type 2, the SFA is partially (type 2a, which is the most common type) or completely absent (type 2b). This case we introduced belongs to type 2a PSA. In types 3 and 4, the SFA are fully developed (1, 2). Type 3 is a partially proximal PSA with a distal deletion, and type 4 is a partially distal PSA with proximal artery absence (3). Gaufre et al. described type 5 PSA, in which the PSA originates from the middle sacral artery; in type 5a, the SFA is complete, and in type 5b, the SFA is absent (8).

While according to the anatomic status and the presence of aneurysm, Ahn et al. proposed a new classification system and treatment option (9). Class I from Ahn-Min's classification includes types 1 and 5a from the Pillet-Gauffre classification, class II includes types 3 and 4, and class III includes types 2a, 2b, and 5b. Class IV is a new class that is not classifiable by the previous Pillet-Gauffre classification. Ahn et al. insist that the risk of embolism from the presence of aneurysm is an important factor for treatment and bypass surgery is mostly required in classes III and IV (9). This case belongs to type 2a according to the Pillet-Gauffre classification, or class III the Ahn-Min's classification, PSA. As PSA was fully present, resulting in adequate distal blood supply and no ischemia or embolism occurred, therefore, conservative treatment was chosen.

PSA can be detected incidentally or confirmed through examinations such as arterial Doppler ultrasound, CT angiography, MRI angiography, or general angiography (10). Colour Doppler ultrasound can reveal gluteal artery aneurysm and determine the presence of a mural thrombus. CT angiography and MRI angiography confirmed the diagnosis and determined its relationship with adjacent anatomical structures, such as the sciatic nerve. Besides, most cases reported in the literature have generally used angiography (11). That's what we did.

The differentiating considerations for pulsatile PSA aneurysms include enlarged bursa, abscess, sciatic nerve hernia, granulomatous disease, tumours, and congenital or acquired arteriovenous fistulas (12).

Treatment depends on the patient's symptoms and relevant anatomical findings, as well as the physiological function and status of the SFA (13, 14). However, femoropopliteal artery bypass grafting is considered inadequate if the common femoral artery is underdeveloped. Therefore, grafting from the PSA (excluding aneurysms) is recommended to avoid persistent exposure of the graft to the hip region due to the potential risk of new aneurysms or pseudoaneurysms in the future, or arterial or graft occlusion (15). When PSA is entangled with the sciatic nerve, endovascular treatment (including a coil or stent graft) can be considered. Future complications such as thromboembolism or rupture of the lower limb should be avoided (4, 8). Asymptomatic, incidentally diagnosed PSA should be treated with medication and regular follow-up, as we have done in the case presented here (16). The patient's lower limb symptoms were attributed to the arteriovenous fistula rather than the persistent sciatic artery, which justified the decision to treat the fistula alone. About the 1-month postoperative drug treatment, we used rivaroxaban 20 mg qd to prevent stent and venous thrombosis, and diosamine 0.9 g bid to improve blood circulation. 1 month after the surgery till now, we administered clopidogrel 75 mg qd as antiplatelet therapy to prevent stent restenosis and thrombotic events in the systemic circulation.

## Conclusions

At the time of the final follow-up(6-month), there was no recurrence of the lower limb discomfort symptoms. The treatment, conservative approach to PSA in this case with iatrogenic femoral arteriovenous fistula, was considered successful in the long term. When choosing the optimal treatment, consideration should be given to whether there is a relationship between lower extremity symptoms and the presence of PSA. Multiple individualised approaches should be considered in future studies. We expect longer follow-up results and more case data.

## Data Availability

The original contributions presented in the study are included in the article/Supplementary Material, further inquiries can be directed to the corresponding author.
